# Psychiatric-psychotherapeutic and psychosocial care for refugees: effects and future prospects of the refuKey project - perspective of experts

**DOI:** 10.1192/j.eurpsy.2022.1633

**Published:** 2022-09-01

**Authors:** N. Wendt, I. Graef-Calliess, K. Loos, D. Finkelstein, V. Mohwinkel, I. Özkan, G. Penteker, B. Trilesnik

**Affiliations:** 1 Netzwerk für Traumatisierte Flüchtlinge e.V., Ntfn, Hannover, Germany; 2 Hannover Medical School, Dept. Of Psychiatry, Hannover, Germany; 3 DTPPP, Transcultural Psychiatry, Psychotherapy And Psychosomatics, Hamm, Germany; 4 Humboldt-Universität zu Berlin, Clinical Psychology, Berlin, Germany

**Keywords:** Refugee, stepped-care, cooperation centers, mental health care

## Abstract

**Introduction:**

Refugees have been shown to be a vulnerable population with increased psychiatric morbidity and lack of access to adequate mental health care. By establishing cooperations between psychosocial centers and psychiatric clinics the state funded project refuKey by NTFN e.V. and DGPPN aims to improve access to and quality of mental health care for traumatized refugees pursuing a stepped-care model.

**Objectives:**

As part of a larger project evaluation study four focus-groups among experts were conducted to explore the impact of refuKey on refugees’ mental health care.

**Methods:**

Data analysis was conducted using Mayring qualitative content analysis as well as an additional quantitative survey with state refugee reception centers’ employees.

**Results:**

The results indicate that refuKey faciliated the access to mental health care for refugees in terms of systematic identification of mental disorders, eased transitions and increased networking between the mental health care institutions and sectors. Planning and implementation of treatment is described as being more coordinated, solution oriented and sustainable due to multiprofessional collaboration and regular use of qualified interpreters. Reduced distress as well as increased transcultural expertise was found for professionals.

**Conclusions:**

The persisting barriers for refugees in access to mental health care, especially to psychotherapeutic treatment and the emotional burden for professionals underlines the need for further support and research. The experts highly endorse the continuance of refuKey. Furthermore, they call for expansion of the project in terms of staff and new sites and changes of health policies to guarantee the access to adequate health care for traumatized refugees.

**Disclosure:**

No significant relationships.

